# Missed nursing care in emergency departments: a cross-sectional descriptive study

**DOI:** 10.1186/s12873-024-00936-9

**Published:** 2024-02-14

**Authors:** Zahra Rooddehghan, Hamid Karimi, Esmaeil Mohammadnejad, Leila Sayadi, Shima Haghani, Raoofeh Karimi

**Affiliations:** 1 School of Nursing and Midwifery, Tehran University of Medical Sciences, Tehran, Iran; 2 Department of Medical-Surgical Nursing, Tehran University of Medical Sciences, Tehran, Iran; 3 Research Center for Antibiotic Stewardship and Antimicrobial Resistance, Tehran University of Medical Sciences, Tehran, Iran; 4 Nursing & Midwifery Care Research Center, School of Nursing and Midwifery, Tehran University of Medical Sciences, Tehran, Iran; 5 Nursing Care Research Center, Iran University Of Medical Sciences, Tehran, Iran

**Keywords:** Missed care, Nursing care, Emergency department, Iran, Frequency

## Abstract

**Background:**

Missed care refers to the omission or delay in performing any aspect of patient’s care (either a part of the care or the entire care). Currently, missed care has become a growing concern at the international level, which threatens the quality and safety of care and cases many unwanted consequences. This study aims to investigate the frequency and types of missed nursing care in the emergency departments of selected hospitals affiliated to Tehran University of Medical Sciences.

**Methods:**

This is a cross-sectional and descriptive- observational study that was conducted with the aim of determining the frequency and types of missed nursing care in the emergency departments of selected hospitals affiliated to Tehran University of Medical Sciences from January 2020 to June 2020. The research community included all nursing care offered in the designated areas, as well as all nurses working in the emergency departments of selected hospitals. Finally, 146 nurses were selected by census method. The information was collected by self-reporting method and the researcher’s observation. Demographic information questionnaire, a researcher-made checklist were used to determine the frequency and types of missed nursing care. 384 observations were made for each item. Descriptive statistics methods were used to analyze the data.

**Results:**

The area of checking equipment and emergency trolley(mean = 81.80) had the lowest and the area of patient communication(mean = 55.72) had the highest level of missed care.

**Conclusions:**

The level of missed nursing care in the emergency departments of selected hospitals affiliated to Tehran University of Medical Sciences was found to be high and the highest amount was related to the field of communication with the patient. Therefore, it is recommended that the details of missed nursing care in each area should be considered by nursing managers.

## Background

Comprehensive nursing care and the type of care that is tailored to the patient’s needs is one of the main goals of nursing, the phenomenon of missed nursing care is still a serious threat to achieving comprehensive and safe nursing care, which also threatens the lives of patients [1].

Missed nursing care is standardly defined as any aspect of care (partial or total) needed by patient, which has been eliminated or significantly delayed [2, 3].

It has been specifically reported that nurse’s job rating is the main predictor of missed care [4]. Also, the nurse’s work environment has been found to be correlated to the missed nursing care [5]. One of the mechanisms that has been proposed for missed care is based on this argument that says when resources are insufficient, nurses are forced to ration their attention among patients and use their clinical judgment to prioritize assessments and interventions, which force them to omit or delay aspects of care that seem unnecessary and this may increase the risk of negative outcomes for patient [5, 6]. Currently, missed care has become a growing concern at the international level, which threatens the quality and safety of care and cases many unwanted consequences [4].

The results of a study showed that most nurses (86%) did not perform one or more aspects of care in their previous shift due to lack of time [7]. The results of a large cross-sectional study on 33,659 nurses at 488 hospitals in 12 European countries also showed that the most missed nursing care included comforting or talking to patients (53%), developing or updating nursing care plans (42%) and providing education to patients and families (41%). In hospitals with a more favorable working environment, the patient-nurse ratio is low and fewer level of missed nursing care is reported [8].

Internationally, the prevalence of missed care in critical care units is high (55–98%) [9, 10]. Meanwhile, the probability of missed nursing care in emergency departments is also high due to the complex and challenging work systems of this department [11, 12]. Several factors, including the large number of patients, shortages of nurses and medical equipment, the presence of acute and life-threatening diseases, and the lack of empty beds for patient admission, make the emergency department susceptible to the occurrence of medical errors [13]. At the same time, the unpredictability of emergency department and the existence of factors such as urgency, unknown patient medical history, and excessive workload add to the vulnerability of patients in emergency departments [14].

Therefore, due to the high statistics of missed nursing care in hospitals, small number of studies conducted on this issue, and lack of accurate information about the exact level, the researchers in this study decided to investigate the frequency and types of missed nursing care in the emergency departments of selected hospitals affiliated to Tehran University of Medical Sciences.

## Methods

### Design and aim

This is a cross-sectional and descriptive-observational study, which was conducted with the aim of determining the frequency and types of missed nursing care in the emergency departments of selected hospitals affiliated to Tehran University of Medical Sciences. This study was carried out from January 2020 to June 2020.

### Study setting and participants

In the current study, the research community included all nursing care offered in the designated areas as well as all nurses working in the emergency department of selected hospitals affiliated to Tehran University of Medical Sciences, including: Imam Khomeini Hospital Complex and Dr. Shariati Hospital. These two hospitals were chosen as the research environment due to their similarities in patient admission, as well as laws and guidelines they use. It should be noted that each of these hospitals has two emergency departments.

### Sample size and power

Since no similar descriptive study was found in emergency departments to investigate the frequency and types of missed care, to determine the minimum sample size required to estimate the frequency of missed nursing care given that the frequency of missed care is calculated based on a score of 0 to 100, at 95% confidence level and accurate estimation of d = 0.05 and standard deviation 25, assuming the prevalence of missed nursing care is 50%, after quantification in the following formula, the minimum required sample size was determined to be 384 observations for each item. The sampling of nursing care was done as an event sampling.

$$n=\frac{{z}_{1-^{\propto }\!\left/ \!_{2}\right.}^{2} pq}{{d}^{2}}=\frac{{1.96}^{2}\times 0.50\times 0.50}{{0.05}^{2}}=384$$

A total of 155 nursing staff, including nursing assistance, nurses with bachelor’s degree, master’s degree and doctorate working as clinical nurses, staff, nursing managers and nursing supervisors, were working at the emergency departments of two selected hospitals (72 people in Shariati Hospital and 83 people in Imam Khomeini Hospital). From these people, 146 nurses who were eligible for the study, were selected by census method. The inclusion criteria were; willing to participate in the study, being a nurse with a bachelor’s degree or higher education working in the emergency department, and having at least six months of clinical work experience in the emergency department. Also, being transferred from the emergency department to another department during the study was among the exclusion criteria.

### Instruments

The data collection in this study was based on the self-reporting method and observation. The following questionnaires and checklist were used to collect the information:

1. Demographic information questionnaire
2. A researcher-made checklist

1. Demographic information questionnaire was used to collect information such as age, gender, marital status, employment status, work experience, education level, type of shift, and work system. This is a researcher-made questionnaire that was completed in-person by the participants.
2. The researcher-made checklist was used to determine the prevalence of missed nursing care. it is an original instrument created by them to determine the frequency and types of missed nursing care. For this purpose, first, a list of emergency department nursing care based on emergency department texts and activities was prepared, before being categorized and each activity being placed in its own category. This checklist had 11 areas, including triage (18 items), physiological monitoring (12 items), implementing orders (10 items), monitoring patient’s condition (10 items), patient communication (6 items), infection control and environmental health (15 items), making decision about patient (15 items), registration (23 items), cardiopulmonary resuscitation (17 items), checking equipment and emergency trolley (8 items) and patient transfer (10 items).

In each area, there are a number of items related to emergency department care. For each item, three options of “done”, “not done” and “not needed” are defined. In each observation, if the desired care was performed, the option is marked by the observer as performed (score 2), if the care was delayed or not performed, the option is marked by the observer as not performed (score 0) and if the care was not necessary, the option is marked by the observer as not required (score 1). The sum of scores in each area is calculated as total score from 0 to 100, with higher score indicating lower level of missed care.

### Validity of the research tools

Validity of the researcher-made checklist and the questionnaire were determined by content validity method, and using the opinions of 10 faculty members at Faculty of Nursing and Midwifery of Tehran University of Medical Sciences as well as the nursing managers and supervisors of the emergency departments at the selected hospitals.

### Reliability of the research tools

Reliability of the researcher-made checklist was determined by two simultaneous observers who were study’s researchers, and the correlation coefficient between the two evaluators was 0.992 for the area of triage, 0.982 for physiological monitoring, 0.983 for implementing orders, 0.993 for monitoring patient’s condition, 0.993 for patient communication, 0.972 for infection control and environmental health, 0.998 for making decision about patient, 0.99 for registration, 0.953 for cardiopulmonary resuscitation, 0.952 for checking equipment and emergency trolley, and 0.972 for patient transfer.

### Data collection

After obtaining the approval of ethics committee and the necessary permits from the relevant officials of the Faculty of Nursing and Midwifery of Tehran University of Medical Sciences, the researcher presented them to the directorates of selected hospitals. Then, by being present in the research environment and after coordinating with the supervisors of emergency departments, the researcher explained the study objectives to the participants and obtained a written consent from them. In order to learn more about the emergency departments, the researcher visited each of the emergency departments of Imam Khomeini Hospital Complex and Shariati Hospital for five days. Demographic information questionnaire was provided to the nurses. Also, in order to collect data regarding the type and frequency of missed nursing care, the researcher completed the checklist form in the morning, evening and night shifts through direct and indirect observation (recording in the report sheet or patient file) during the weekdays. The observation method was collaborative in such a way that the nurses under study were aware of the presence of the researcher and the researcher helped them in order to cooperate more in carrying out some care activities. First, the nurses’ work list was checked and the clinical nurses were divided into three equal groups, so that the number of care delivery observed in the morning, evening and night shifts would be equal with a slight difference. A code was assigned to each nurse in each hospital and the defined code was attached to the checklist. The researcher completed the checklist for each code equal to the number of beds that the nurse of that shift was responsible for, by observing him/her. For observation, first, the nurses’ care in the morning shift was checked and the relevant checklist was completed for each nurse according to the number of beds she/he was responsible for. In order to increase the accuracy of the survey, we tried not to have more than two nurses under observation each time. In the same way, in the evening and night shifts, the corresponding checklist was completed for each nurse. It should be noted that the observation and completion of the checklists were done alternately by the researcher and a second person who was at the same level as the researcher in terms of university education. In order to establish coordination between the two observers, the work process, observation framework and checklist items were reviewed and corrected by them before starting the work and completing the checklists.

### Statistical analysis

Data analysis was done by SPSS software version 16. Frequency distribution tables were used for qualitative variables, and minimum, maximum, mean and standard deviation numerical indicators were used for quantitative variables.

### Ethical considerations

This study was conducted by obtaining the code of ethics (IR.TUMS.FNM.REC.1398.109) from the joint ethical committees of the Nursing & Midwifery and Rehabilitation Faculties of Tehran University of Medical Sciences. Informed consent was taken from the participants or their guardians prior to their participation in the study. The privacy of study subjects was maintained, or informed consent was obtained if they were in any way identifiable.

## Results

In total, out of 155 personnel who worked in the emergency departments of these two hospitals, 6 nursing assistance at Imam Khomeini Hospital (due to not having the entry criteria of having at least a bachelor’s degree in nursing), one nurse (due to not having at least six months of clinical experience in the emergency department) and two more nursing assistance at Shariati Hospital were excluded from the study. Finally, 146 eligible people were included in the study. After sampling, no sample drop was observed during the study and all the samples completed the questionnaires.

The frequency distribution of demographic characteristics of emergency department nurses can be seen in Table 1.

**Table 1:** Frequency distribution of individual characteristics of nurses in emergency departments of selected hospitals affiliated to Tehran University of Medical Sciences – 2020

Individual characteristics		Frequency	percentage	Standard deviation ± mean, maximum-minimum
Age (years)	Less than 30	61	41.8	32.5 ± 29.95 49–23
	39–30	62	42.5	
	40 and above	23	15.8	
	Total	146	100	
Gender	Female	106	72.6	
	Male	40	27.4	
	Total	146	100	
Hospital	Imam Khomeini	76	52.1	
	Shariati	70	47.9	
	Total	146	100	
Position	Clinical nurse	136	93.2	
	Staff	1	0.7	
	Nurse manager	3	2.1	
	Supervisor	6	4.1	
	Total	146	100	
Marital status	Single	56	38.4	
	Married	88	60.2	
	Other	2	1.4	
	Total	146	100	
Employment status	Official	48	32.9	
	Full time	32	21.9	
	Contractual	13	8.2	
	Supernumerary	18	12.3	
	Corporate	35	24	
	Total	146	100	
Type of work shift	Morning	11	7.5	
	Evening	6	4.1	
	Night	29	19.9	
	Morning and evening	39	26.7	
	Evening and night	24	16.4	
	Circulatory	37	25.3	
	Total	146	100	
Education level	Bachelor’s degree	131	89.7	
	Master’s degree	9	6.2	
	Postgraduate student	5	3.4	
	Doctorate	1	0.7	
	Total	146	100	
Work experience as a nurse (year)	Less than 5	44	30.1	8.5 ± 43.69 0.5–27 (years)
	5–9	49	33.6	
	10–15	32	21.9	
	Above 15	21	14.4	
	Total	146	100	
Work experience in emergency department (year)	Less than 5	70	47.9	5.4 ± 98.62 0.5–27 (years)
	5–10	42	28.8	
	Above 10	34	23.3	
	Total	146	100	
Employment in other hospitals	Yes	96	65.8	
	No	50	34.2	
	total	146	100	

The results of Table 2 show that the following activates are the most missed nursing care among the participants in this study:

**Table 2:** Frequency distribution of missed nursing care in different areas in the emergency departments of selected hospitals of Tehran University of Medical Sciences-2020

**Areas**^**b**^			**Total number of observations**^**c**^	**Done**^**d**^** (1)**	**Not done**^**e**^** (0)**	**Not necessary**^**f**^** (1)**	**Mean (SD**^a^)
	**No**	**Items**		**Frequency (%)**	**Frequency (%)**	**Frequency (%)**	
The area of triage^g^	1	It is performed by a nursing expert with at least 5 years of clinical work experience, of which at least one year is in the emergency department^g^.	384	384 (100)	0 (0)	0 (0)	2 (0)
	2	It takes place upon arrival to emergency department and even before admission and payment.	384	384 (100)	0 (0)	0 0	2 (0)
	3	The triage nurse’s evaluation of second level patient is done less than 5 min after the patient’s entry to emergency room	384	31 (8.1)	26 (6.8)	327 (85.2)	1.01 (0.38)
The area of physiological monitoring	1	Blood pressure is controlled	384	281 (37.2)	84 (21.9)	19 (4.9)	1.51 (0.83)
	2	The temperature is measured	384	209 (54.4)	164 (42.7)	11 (2.9)	1.11 (0.97)
The area of implementing orders	1	Requested tests are sent	384	280 (72.9)	73 (19)	31 (8.1)	1.53 (0.79)
	2	When giving medication to patients, the correct medication process is observed	384	172 (44.8)	212 (55.2)	0 (0)	0.89 (0.99)
The area of patient’s condition	1	When the patient is in bed, the bedrails are up	384	184 (47.9)	198 (51.6)	2 (0.5)	0.96 (0.99)
	2	The results of tests are followed up	384	325 (84.6)	57 (14.9)	2 (0.5)	1.69 (0.71)
The area of patient communication	1	The nurse establishes effective and reassuring communication with the patient	384	189 (49.2)	195 (50.8)	0 (0)	0.89 (1)
	2	The nurse teaches the patient the minimum items to be taught to the patient during hospitalization	384	199 (51.8)	116 (30.2)	69 (18)	1.21 (0.88)
The area of infection control and environmental health	1	After contact with body fluids and secretions of the patient, the nurse washes her hands	384	358 (93.2)	26 (6.8)	0 (0)	1.86 (0.5)
	2	The nurse observes the principles of sterilization in sterile procedures	384	169 (44)	203 (52.9)	12 (3.1)	0.91 (0.98)
The area of making decision about patient	1	The nurse performs the necessary follow-up to determine the patient’s condition	384	301 (78.4)	80 (20.8)	3 (0.8)	1.57 (0.81)
	2	Proper coverage and privacy of the patient is observed during the transfer	384	292 (76)	71 (18.5)	21 (5.5)	1.57 (0.78)
	3	The schedule informs the patient/family about the scheduled visits after discharge	384	89 (23.2)	80 (20.8)	215 (56)	1.02 (0.66)
The area of registration	1	The nursing report is recorded with a blue or black pen	384	377 (98.2)	7 (1.8)	0 (0)	1.96 (0.26)
	2	No nursing action is recorded before it is performed	384	94 (24.5)	290 (75.5)	0 (0)	0.48 (0.86)
The area of cardiopulmonary resuscitation	1	The team/resuscitation room nurse is aware of her roles and duties and is actively present	384	297 (77.3)	57 (14.8)	30 (7.8)	1.62 (0.72)
	2	The nurse of the resuscitation team has attached the resuscitation code card, which shows the description of duties, to the patient’s chest	384	44 (11.5)	114 (29.7)	226 (58.9)	0.81 (0.61)
The area of checking equipment and emergency trolley	1	The emergency trolley is continuously managed and updated by the responsible nurse in each work shift	384	180 (46.9)	77 (20.1)	127 (33.1)	1.26 (0.77)
	2	The arrangement of medications and equipment in the resuscitation trolley is done according to the latest instructions issued by the Ministry of Health	384	384 (100)	0 (0)	0 (0)	2 (0)
	3	It is possible to have easy, immediate and unhindered access to the resuscitation trolley within a minute	384	384 (100)	0 (0)	0 (0)	2 (0)
The area of patient transfer	1	The patient’s identification wristband and all treatment accessories are checked by the nurse in terms of correctness	384	187 (48.7)	170 (44.3)	27 (7)	1.04 (0.96)
	2	The nurse describes the current condition of the patient within 5 to 10 s	384	313 (81.5)	71 (18.5)	0 (0)	1.63(0.77)

^a^*SD* Standard Deviation

^b^It represents all the work areas that exist in the emergency department (11 areas) and each area includes several activities, in this table only the most and the least missed nursing care in that work area is stated

^c^It shows the number of times each care was observed in each area in the emergency department

^d^Number of times each care was performed and not missed

^e^Number of times each care was missed

^f^The number of times that it was not necessary to perform this care

^g^For example, this area has been observed 384 times, and regarding activities “It is performed by a nursing expert with at least 5 years of clinical work experience, of which at least one year is in the emergency department” and “It takes place upon arrival to emergency department and even before admission and payment.” 384 times out of 384 observations were made and not missed, 26 times out of 384 observations were missed in” The triage nurse’s evaluation of second level patient is done less than 5 min after the patient’s entry to emergency room”

The triage nurse’s assessment of level two patient in less than 5 min of patient’s entry to the emergency room in the area of triage(mean = 1.01), the temperature measurement in the area of physiological monitoring(mean = 1.11), the compliance with the correct drug therapy in the area of implementing orders(mean = 0.89), the bedrails are up when the patient is in the bed in the area of monitoring patient’s condition(mean = 0.96), establishing effective and reassuring communication with the patient in the area of patient communication(mean = 0.98), observing the principles of sterilization in sterile procedures in the area of infection control and environmental health(mean = 0.91), giving the information of schedule visits after discharge to patient or family in the area of making decision about patient(mean = 1.02), no nursing care is recorded before it is performed in the area of registration(mean = 0.48), the nursing member of resuscitation team attaches the card of resuscitation code, which indicates the description of duties, to the chest in the area of cardiopulmonary resuscitation(mean = 0.81), the emergency trolley is checked and resupplied by the nurse at the beginning of each shift in the area of checking equipment and emergency trolley(mean = 1.26), and the patient’s identification wristband and all the treatment accessories are checked by the nurse at the next shift in terms of correctness in the area of patient transfer(mean = 1.04).

The area of checking equipment and emergency trolley and the area of patient communication obtained the highest and lowest mean score (80.81 and 55.72), respectively (Table 3).

**Table 3:** Numerical indicators of the areas of missed nursing care in the emergency departments of selected hospitals affiliated to Tehran University of Medical Sciences – 2020^b^

The areas of missed nursing care	Minimum	Maximum	Mean	SD^a^
Triage	36.11	88.89	72.39	13.21
Physiological monitoring	20.83	100	68.17	16.99
Implementing orders	25	100	65.01	14.25
Monitoring the patient’s condition	35	100	69.85	15.48
Patient communication	0	100	55.72	24.01
Infection control and environmental health	33.33	100	62.50	11.70
Making decision about patients	26.67	96.67	64.34	12.46
Registration	50	97.83	76.07	9.96
Cardiopulmonary resuscitation	20.59	100	59.15	11.65
Checking equipment and emergency trolley	25	100	80.81	15.66
Patient transfer	30	100	71.26	14.32

^a^*SD* Standard Deviation

^b^It shows the highest average of care being performed and the lowest average of care not being performed, so the area of communication with the patient has the highest and check trolley and equipment has the lowest amount of missed care

## Discussion

The present study was conducted in order to determine the frequency and types of missed nursing care in the emergency departments of selected hospitals affiliated to Tehran University of Medical Sciences. The results showed that the lowest and highest level of missed nursing care was observed in the areas of checking equipment and emergency trolley, and patient communication, respectively.

### The area of triage

The triage nurse’s decisions directly affect the time of service delivery, and errors in patient triage have serious consequences. The Adjunct Committee on Fatalities Report states that half of all accidents that result in death occur as a result of delayed treatment in the emergency department [15].

In a medical emergency, seconds and minutes are important for the patient, and these times can determine the death, serious disability or life of patient [16].

In line with the current study, Kamrani et al. (2013) believed that the triage error rate is high, so more serious training is needed to improve the ability of nurses to accurately identify and classify high-risk patients referred to the emergency department [17].

### The area of physiological monitoring

The findings of present study are consistent with the results of Saki et al. [18] study that investigated the rate of nursing errors in emergency department and its relationship with different dimensions of fatigue. They found that, one of the areas of error was related to the control of vital signs. However, among the 4 items related to this area, the item of “carelessness in taking respiratory rate” had the highest error rate followed by the items of “carelessness in taking the heart rate”, “carelessness in taking the temperature” and “carelessness in taking blood pressure”. In the present study, temperature measurement in the field of physiological monitoring accounted for the highest level of missed nursing care.

### The area of implementing orders

In line with the results of present study, Bayat Menesh et al. [19] in a study that observed the performance of nurses working in three ICUs(Intensive Care Unit) of selected hospitals affiliated to Yasouj University of Medical Sciences, found that the safety principles related to correct drug therapy were not fully observed and were at an unfavorable level [19]. Also, the results of Mozafari et al. [20] study, which examined the amount of mistakes in the implementation of medication orders in the general departments of hospitals in Ilam city, showed that the most types of medication errors in the field of oral medications were related to not washing hands, not controlling the expiry date of drugs, and not checking the type and dose of drugs with the patient’s drug chart. In the field of injectable drugs, the most medication errors were related to the lack of necessary training to patient in relation to the prescribed drugs, lack of training to patient and patient’s companion in relation to the manipulation of IV(Intra Venous) serum and lack of staff’s handwashing. Their findings also showed that both in the field of oral drugs and injectable drugs, most of the nurses did not provide the necessary training related to medication to patients, which could be attributed to the nurses’ lack of information on drugs and their side effects, high workload, shortages of staff and physical or mental fatigue [20].

### The area of monitoring patient’s condition

A significant number of patients admitted to emergency department are at risk of falling [21]. Consistent with the present study, a cross-sectional study on 300 nurses working in the hospitals of Tehran University of Medical Sciences showed that the average exposure to patient fall in the last 3 months was 6 ± 1.61 times for each nurse. Also, the highest number of falls was reported for falling from bed while resting (8.18 ± 0.47) and falling while walking (7.26 ± 0.44) [22]. Min et al. [23] conducted a cross-sectional study on the relationship between nurses’ rest, missed nursing care and patient safety in Korean hospitals through an online survey, according to which the most missed nursing care included; completing documents, emotional support, handwashing, and attending meetings. Also, the most causes of missed nursing care were related to human resources, material resources and communication. These results are in line with the findings of present study. The results of Min et al. [23] study also showed that the frequency of missed nursing care was significantly related to patient safety, medication errors, falls and injuries, and also the number of patients assigned to each nurse was directly correlated to patient safety, injury and falls [23].

### The area of patient communication

The findings of Khorasani Zavareh et al. [24] study showed that patients expect nurses to regularly visit them during their stay in the emergency department and check their condition. Patients also expect nurses to express sympathy and empathy towards them [24], which is in line with the present study. Lotfi et al. [25] conducted a study entitled: “Evaluation of nurse-patient relationship and patient satisfaction with nursing care in Iran”. In this study, 295 patients were investigated, and the most dissatisfaction of patients with nursing care included; “not taking into account my opinions and preferences about my care plans”, “it should be more precise”, “neglect about making sure that I understand the importance of treatment”, and “making me feel like I’m a case, not a person” [25].

### The area of infection control and environmental health

The results of present study are in line with the findings of Ziasheikholeslami et al. study, which was conducted on the ICU staff of a hospital in Qom. In this study, the highest rate of handwashing (96%) was done after exposure to patient’s blood and body fluids, after contact with the surrounding environment of the patient (61.9%), after contact with the patient (61.36%), before the aseptic procedure (27.27%) and finally, before contact with the patient (3.38%) [26]. Handwashing is critical in emergency departments, where essential treatments often involve high-risk and invasive procedures, and there is no time left to assess the patient’s susceptibility to infection [27]. General factors affecting hand hygiene and infection control in emergency departments include workplace culture, high speed of actions required in emergency cases, frequent interruptions, heavy workload, lack of time, prioritizing patients’ needs over hand hygiene, allocating patients in non-medical areas such as corridors, access to facilities and products, and overcrowding [28].

### The area of making decision about patients

Increasing the length of stay in emergency department and not making decision about patients in a timely manner have a negative impact on the quality of service delivery, increase dissatisfaction and eventually promote violence. According to the findings of Nasra Asfahani and colleagues, one of the common causes of long patient stay in the emergency department is the delay in making decision about patient by other services [29]. Meanwhile in the present study, the item “informing patient or family about the schedule of planned visits after discharge” was the most missed care in the area of making decision about patient, which is related to the discharge process. Busy work and lack of proper coordination between physicians and nurses are among factors that contribute to the non-compliance with the discharge instructions and informing patient about next visit schedule.

### The area of registration

According to the findings of Esmailian et al. [30] study in Al-Zahra Hospital in Isfahan, the status and quality of meeting the standards of documentation in the emergency department is not at favorable level. Thus, the highest documentation defects in nursing reports is related to not closing the end of report with a straight line, not mentioning the reason, method and type of referral upon arrival, and not providing sufficient explanations about the general condition of patient. Meanwhile in the present study, the most missed nursing care in the area of registration was related to the item of “no nursing action is recorded before it is performed”. Considering the importance of nursing reports and their legal role, nurses’ lack of awareness about legal issues, high workload, and lack of sufficient supervision and control are among factors that contribute to nurses’ reporting and recording actions before doing them. In Iranian hospitals, due to legal issues, patient information must be recorded in two electronic and paper ways, because electronic reports are not considered legal documents in the eyes of judicial authorities, and this causes an increase in the workload and rework of nurses that result in the increased complaints and dissatisfaction of nurses [31].

### The area of cardiopulmonary resuscitation

Based on the findings of Kavosi et al. [32] study, the most important obstacles to the success of effective cardiopulmonary resuscitation from the nurses’ point of view included patients’ underlying disease (88%), lack of timely presence of resuscitation team at the patient bedside (98%), lack of effective communication skills among team members (90%), lack of facilities and CPR(Cardio Pulmonary Resuscitation) equipment in each department (92%), and finally, lack of regular standard and basic in-service training (98%) [32]. However, in the present study, the reasons for the failure of cardiopulmonary resuscitation were not investigated or considered, while the evaluation of missed nursing care in the area of cardiopulmonary resuscitation showed that the item; “the nursing member of resuscitation teem is aware of his/her roles and duties and has an active presence” accounted for the lowest missed nursing care in this area.

### The area of checking equipment and emergency trolley

Smith et al. [33] conducted a study in UK entitled: “Re-stocking resuscitation trolley: How good is compliance with checking procedures”? The purpose of this study was to retrospectively examine the methods of checking resuscitation trolleys in medical, surgical and pediatric wards to evaluate compliance with checking procedures. The average level of checking in the medical and surgical wards was 72.2 and 68.8%, respectively. Hospital policy states that on these trolleys, the equipment must be checked at the beginning of each nursing shift (twice a day), but the resuscitation trolley equipment was checked more than once a day on 12 days (3.3%) of the year in the medical ward and 16 days (4.4%) of the year in the surgical ward. Also, the results of this study showed that in the relevant hospital, the basic methods of checking trolleys were not followed, nurses probably did not prepare the trolley for an emergency cardiopulmonary arrest, and even a trolley could remain unchecked for up to 9 days. Also, trolleys were found to be checked more often during the night shift, which was likely due to low level of workload at that time. Which is in line with the present study.

### The area of patient transfer

Nursing handover is an important part of care provision, where the care responsibilities and duties are transferred from one nurse to another nurse. According to the present study, the item of “patient’s identification wristband and all treatment accessories are checked by the nurse after the shift in terms of correctness” accounted for the highest level of missed nursing care in the area of patient transfer. In this regard, it seems that the high workload, high patient/nurse ratio, lack of sufficient supervision in this field, especially in the evening and night shifts, and the friendly relationship between nurses are among the factors that can affect the accuracy of information exchange and the comprehensive examination of patients during shift handover. In their study, Baghaei et al. [34] concluded that the transfer of information and patient in the form of standard shift handover guide such as the SBAR(Situation-Background-Assessment-Recommendation) model is effective in the patients’ view of nursing care quality [34].

## Conclusions

Results of present study showed that the level of missed nursing care in emergency departments was high, and the highest level of missed nursing care was related to the area of patient communication. Knowing these factors is useful and practical for nursing managers and nurses, as it helps them to prepare and implement the necessary measures and solutions to improve the quality of nursing services in emergency departments.

### Strengths and limitations

Since 2006, many studies have been conducted on missed nursing care and its causes. However, this issue has not been investigated in the emergency department so far, and the related studies have only investigated this issue through questionnaires, while in this study, this issue was investigated by being in the field and observing the nurses’ performance directly. Although being observed is one of the problems of all observational studies that can overshadow the behavior of research samples, to control this issue, the frequent presence of researcher in different shifts, establishing intimate relationship with the staff and sometimes participating in the nursing activities assigned to the research samples limited this problem.

## Data Availability

The datasets are available from the corresponding author on reasonable request.

## Abbreviations

ICU: Intensive Care Unit
IV: Intra Venous
CPR: Cardio Pulmonary Resuscitation
SBR: Situation-Background-Assessment-Recommendation
